# Tannin; Its Use in Pruritus Vulva and Vagina

**Published:** 1865-12

**Authors:** 


### TANNIN; ITS USE IN PRURITUS VULVA AND VAGINA.

This distressing and not infrequent affection, has been more or less elaborately treated upon in all our standard works upon gynecology, yet the intractable nature which it sometimes assumes, proving rebellious to approved medication, imperiously demands that anything capable of impressing a remedial influence upon it, should not go unmentioned, which is my apology for proposing the addition of Tannin to the armamentarium of remedies for this disease.

There are probably few diseases more distressing than the one under consideration, causing, as it sometimes does, mental depression, nymphomania, and consequent seclusion from society. Yet in some of the most intractable cases that have ever come under my care, the tannin has effected a cure, after failure with the most approved remedies, and when the condition of the patient was pitiable in the extreme. In one intractable case of two years duration, which came under my care, after having undergone a variety of treatment from two excellent physicians, the tannin succeeded perfectly, after failure with Prof. Meig's recipe, and other standard remedies.

In idiopathic pruritus vulva and vagina, my experience would lead me to regard it as much a specific as quinia for intermittents, or sulphur for the acarus scabiei. In the symptomatic variety, its remedial agency is less decided, but far superior to that of any other agent that I have ever employed. Here, of course, the remote cause must be sought for and appropriately treated as far as practicable, be it disease of the uterus or its appendages, ascarides in the rectum, vesical irritation, or whatever it may be, lest a recurrence of the disease take place.

I usually employ the following recipe: ℞. Acidi Tannici, ʒ i.; Adepis, ℥ i. Fiat unguentum. To be applied twice or thrice daily, to the diseased surface, or as an extemporaneous prescription of a teaspoonful of powdered tannin, to a tablespoonful of lard. Should it produce smarting, which it rarely does, the strength of the recipe should be reduced.

When the disease is symptomatic of uterine disease, in addition to the local treatment advocated by our gynecologists, I can confidently recommend the following prescription as an efficient alterative: Finely ground Helonias Dioica, ℥ ii.; finely ground Cimicifuga Racemosa, ʒ i.; Senecio Aureus, ʒ i. Digest for fourteen days in a quart of good wine or whiskey. Dose from a half to a table spoonful three times a day, preferably given after meals.

Should the patient be anemic, some chalybeate should be exhibited. Of the different chalybeate preparations, Squibbs' pyrophosphate of iron commends itself to our confidence by its solubility, almost tastelessness, and general acceptability to the stomach.

---

We would request the writer of the above article to send us his name and address, as we have unfortunately lost, if we have ever received them.—EDS. JOURNAL.

